# Dietary fiber concentration modulates the efficacy of phytogenic and antimicrobial rumen modifiers on ruminal fermentation and methane emissions in cattle

**DOI:** 10.1016/j.vas.2026.100785

**Published:** 2026-07-27

**Authors:** Geraldo Balieiro Neto, Alexandre Berndt, Cristina Maria Pacheco Barbosa, Carlos Eduardo Kolb Maynardes Araujo de Campos Jordao, Gabriela Aferri, Leandro Samia Lopes

**Affiliations:** a Animal Science Institute, Department of Agriculture and Food Supply of São Paulo State Government (IZ-SAA), Ribeirão Preto, São Paulo, Brazil; b Brazilian Agricultural Research Corporation Southeast Livestock (EMBRAPA), São Carlos, São Paulo, Brazil; c Animal Science Institute, Department of Agriculture and Food Supply of São Paulo State Government (IZ-SAA), Nova Odessa, São Paulo, Brazil; d Animal Science Department (DZO), Federal University of Lavras (UFLA), Lavras, Minas Gerais, Brazil

**Keywords:** Ammonia nitrogen, Essential oils, Feeding behavior, Methane emissions, Short-chain fatty acids, Rumen modulation

## Abstract

This study evaluated the effects of dietary fiber level and rumen modulators on feeding behavior, methane emissions, and ruminal fermentation parameters in cattle. A single 4 × 4 Latin square design with a 2 × 2 factorial arrangement of treatments was used, involving four ruminally cannulated cattle evaluated over four experimental periods. The treatments combined two dietary neutral detergent fiber (NDF) concentrations (12% and 50% of dry matter) with two feed additive strategies: monensin plus virginiamycin (VM) or a phytogenic additive (LM+). Diets containing 12% NDF represented forage-free conditions, whereas diets containing 50% NDF represented conventional fiber-based feeding systems. Dry matter intake, rumination time, ruminal pH, and liquid passage rate were greater in animals fed 50% NDF diets (P < 0.05). Methane emissions were substantially higher in 50% NDF diets, averaging 153.1 versus 46.9 g/day, and 12.8 versus 8.8 g/kg DMI for 50% and 12% NDF diets, respectively. Within the 50% NDF diets, LM+ reduced methane emissions by 26% compared with VM (130.0 vs. 176.3 g/day; P < 0.05). Within the 12% NDF diets, LM+ increased total short-chain fatty acid concentrations by 34% compared with VM (95.0 vs. 70.9 mM) and increased butyrate concentration (20.2 vs. 11.9 mM), whereas VM reduced ruminal ammonia nitrogen concentration (12.4 vs. 16.9 mM; *P* < 0.05). Lactate concentrations were markedly greater in 12% than in 50% NDF diets (10.7 vs. 1.3 mM; P < 0.05), regardless of additive. Dietary NDF concentration was the major factor influencing ruminal fermentation, feeding behavior, and methane emissions, although several responses depended on diet × additive interactions. These results indicate that the efficacy of phytogenic and conventional antimicrobial rumen modulators is strongly dependent on dietary fiber concentration and the prevailing ruminal fermentation environment.

## 1 Introduction

High-concentrate feeding systems have been increasingly adopted in intensive ruminant production because of their ability to increase dietary energy density and modify ruminal fermentation patterns. Reductions in dietary fiber concentration markedly alter ruminal microbial ecology by increasing the availability of rapidly fermentable carbohydrates, which directly influences short-chain fatty acid production, ammonia metabolism, ruminal pH, and methane emissions (Plaizier et al., 2017). Under these conditions, changes in ruminal fermentation may also affect feeding behavior and ruminal kinetics.

Among feed additives used in intensive feeding systems, ionophores such as monensin are widely applied because of their ability to improve feed efficiency and modulate ruminal fermentation (Dinius et al., 1976; Richardson et al., 1976; Russell & Strobel, 1989; Thornton & Owens, 1981). In addition, non-ionophore antimicrobials such as virginiamycin are commonly included in high-concentrate diets to improve ruminal stability and reduce the risk of digestive disorders. These additives are frequently associated with reductions in the acetate-to-propionate ratio, ruminal ammonia nitrogen concentration, and methane production, alongside improvements in nutrient utilization efficiency (Russell & Strobel, 1989). In high-concentrate feeding conditions, antimicrobial additives may also contribute to ruminal stability by inhibiting Gram-positive bacteria involved in excessive lactate production (Dennis et al., 1981; Nagaraja & Lechtenberg, 2007; Nagaraja et al., 1982; Nagaraja et al., 1997).

Interest in alternative rumen modulators has increased in recent years because of concerns regarding antimicrobial use in livestock production and the search for nutritional strategies capable of modulating ruminal fermentation through non-antibiotic mechanisms (Dorantes-Iturbide et al., 2022). Among these alternatives, phytogenic additives containing essential oils and other plant-derived secondary metabolites have received increasing attention because of their antimicrobial properties and their potential to influence ruminal microbial activity and fermentation pathways (Benchaar et al., 2008; Burt, 2004).

Previous studies evaluating phytogenic additives have reported variable effects on feed efficiency, ammonia metabolism, volatile fatty acid production, and methane mitigation (Cobellis et al., 2016; Hristov et al., 2013; Olijhoek et al., 2021). Such inconsistencies suggest that the response to these additives is strongly dependent on dietary composition, particularly fiber concentration and the availability of fermentable carbohydrates. In high-concentrate diets, rapid starch fermentation may substantially modify ruminal conditions and alter microbial responses to rumen modulators.

Recently, a phytogenic additive containing essential oils and rumen-modulating compounds was evaluated in cattle fed forage-free diets, resulting in alterations in ruminal fermentation and productive responses (Ovani et al., 2026). However, methane emissions were not evaluated, and the response of phytogenic additives across feeding systems differing markedly in fiber concentration remains poorly understood. The present study expands upon those findings by evaluating the same phytogenic additive under contrasting dietary NDF concentrations while simultaneously assessing methane emissions, ruminal kinetics, and fermentation responses. Little information is available regarding whether the efficacy of phytogenic additives differs between forage-free and fiber-based feeding systems, particularly when methane emissions, ruminal kinetics, and fermentation characteristics are evaluated simultaneously.

In the present study, ruminally cannulated cattle were used as an experimental model to evaluate fermentation patterns, methane emissions, feeding behavior, and ruminal parameters under controlled dietary conditions. This approach allowed repeated measurements of ruminal responses associated with contrasting dietary fiber concentrations and different rumen modulators. Therefore, this study aimed to evaluate the effects of a phytogenic additive compared with a conventional antimicrobial additive program based on monensin and virginiamycin in diets containing contrasting neutral detergent fiber concentrations on feeding behavior, ruminal fermentation, protozoal populations, ruminal kinetics, and enteric methane emissions. We hypothesized that reducing dietary NDF would alter the efficacy of rumen modulators by modifying the ruminal microbial ecosystem, resulting in distinct patterns of SCFA production, nitrogen metabolism, and methane emissions.

## 2 Materials and methods

### 2.1 Experimental design, animals, and diets

The study was conducted at the Experimental Farm of the Instituto de Zootecnia, Ribeirão Preto, São Paulo, Brazil, in accordance with the guidelines of the Animal Ethics Committee of the State of São Paulo (CEUA/IZ/APTA/SAA; protocol 402-2023). A single 4 × 4 Latin square design with a 2 × 2 factorial arrangement of treatments was used to evaluate the effects of dietary NDF concentration and rumen modulator strategy. Four ruminally cannulated multiparous cattle (3/4 to 7/8 Holstein × Gir; >3 parities; body weight: 585 ± 25 kg) were used as the experimental units. Each animal received each of the four treatments once over four experimental periods, resulting in four observations per treatment.

The experimental factors consisted of two dietary neutral detergent fiber (NDF) concentrations and two rumen modulator strategies. Diets contained either 12% or 50% NDF (dry matter basis), representing forage-free and conventional fiber-based feeding systems, respectively (Plaizier et al., 2017). The 50% NDF diet consisted of a forage-based ration, whereas the 12% NDF diet was formulated to increase dietary energy density and simulate a forage-free, high-concentrate feeding system.

The forage-based diet included ground *Brachiaria* spp. hay and concentrate ingredients (corn, soybean meal, urea, and mineral premix). The forage-free diet consisted primarily of whole corn grain and a protein-vitamin-mineral pellet. Ingredient and chemical compositions of the experimental diets are presented in Table 1. The pellet included the phytogenic additive (LM+), composed of essential oils and rumen-modulating compounds derived from garlic (*Allium sativum*), eucalyptus (*Eucalyptus spp.*), oregano (*Origanum vulgare*), neem (*Azadirachta indica*), yucca (*Yucca schidigera*), and annatto (*Bixa orellana*), in addition to tannins, and nucleotides (Benchaar et al., 2008; Dorantes-Iturbide et al., 2022; Ovani et al., 2026).

**Table 1:** Ingredient and chemical composition of experimental diets.

	__ Experimental diets ___			
	__ _50% NDF __		___ 12% NDF ___	
Ingredients	VM	LM+	VM	LM+
Forage^a^	50	50	0	0
Protein-mineral pellet^b^	-	15	-	15
Concentrate^c^	50	35	100	85
Chemical composition (g kg^-1^ DM)				
Crude protein	155	156	147	141
Neutral detergent fiber	518	485	128	123
Acid detergent fiber	326	300	40	30
Total Digestible Nutrients (TDN)	699	696	847	849
Lignin	54	46	0	0
Ether extract	36	35	39	39
Starch	214	232	546	561
Ash	58	78	42	48

a *Brachiaria spp. hay.*

b Protein-vitamin-mineral pellet including Factor LM+, a proprietary additive based on essential oils and rumen-modulating properties

c The concentrate was composed of 85.0 % ground corn, 8.4 % soybean meal, 0.9 % urea, and 5.7 % mineral premix.

The rumen modulator strategies consisted of supplementation with monensin (ionophore) plus virginiamycin (VM) (Dinius et al., 1976; Richardson et al., 1976; Russell & Strobel, 1989) or the phytogenic additive (LM+), evaluated as an alternative rumen-modulating strategy. Monensin and virginiamycin were included at 22 mg/kg dietary dry matter each, whereas the phytogenic additive (LM+) was included at 7.5 g/kg dietary dry matter. Diets were formulated to meet the nutritional requirements of cattle under intensive feeding conditions and were offered as total mixed rations once daily at 08:00 h. Animals were fed ad libitum with approximately 5–10% orts.

### 2.2 Experimental period and intake measurements

The experiment lasted 96 days, divided into four 24-day periods. The first 14 days of each period were used for dietary adaptation, allowing animals to adjust to the experimental diets and stabilization of ruminal fermentation patterns (Salfer et al., 2018). Previous studies in dairy cows have demonstrated that ruminal fermentation and digestive responses undergo substantial adjustments within the first weeks following dietary changes (Matthé et al., 2003). Methane measurements were conducted from day 15 to day 21 of each experimental period. Ruminal fluid sampling for fermentation analyses was performed from day 21 to day 23, and ruminal liquid kinetics was evaluated on day 24. Dry matter intake (DMI) was calculated daily as the difference between feed offered and orts collected.

### 2.3 Methane emissions

Enteric methane emissions were measured using the sulfur hexafluoride (SF₆) tracer technique (Berndt et al., 2014). SF₆ permeation tubes were placed in the rumen 15 days before measurements and remained in situ throughout the experiment. Gas sampling was conducted over 7 consecutive days per period, with continuous 24 h collection. Methane emission rates were calculated from CH₄ and SF₆ ratios, corrected using background air samples.

### 2.4 Feeding behavior

Feeding behavior was evaluated by continuous 24 h visual observation on day 20 of each period. Activities were classified as eating, rumination, or idle, and expressed as min/day (Galyean & Defoor, 2003).

### 2.5 Ruminal sampling and chemical analyses

Ruminal fluid was collected via the cannula at 0 (immediately before the morning feeding at 08:00 h), 1, 2, 4, and 8 h after feeding. Approximately 500 mL of ruminal fluid was collected manually from each animal, strained through two layers of cheesecloth, and immediately transported to the laboratory. After subsampling, the remaining ruminal contents were returned to the rumen. For short-chain fatty acid (SCFA) analysis, approximately 100 mL of ruminal fluid was centrifuged at 3500 rpm for 15 min, and 1 mL of the supernatant was transferred to a sealed tube containing 0.2 mL of analytical-grade formic acid and stored at −20°C until analysis. SCFA concentrations were determined by gas chromatography according to the method of Erwin et al. (1961) using a gas chromatograph (Model 9001, Finnigan, USA) equipped with an 80/120 Carbopack™ B-DA/4% Carbowax® 20M packed column. Injector, column, and flame ionization detector temperatures were maintained at 195, 120, and 205°C, respectively. Samples (1 µL) were analyzed against external standards containing acetate, propionate, butyrate, isobutyrate, 2-methylbutyrate, valerate, and isovalerate. Each sample was analyzed in duplicate, and additional injections were performed whenever the difference between duplicate determinations exceeded 5%. Ammonia nitrogen was analyzed using the phenol-hypochlorite method (Broderick & Kang, 1980). Lactate was determined spectrophotometrically (Pryce, 1969). Protozoa were quantified using a Neubauer chamber following Dehority (1984).

### 2.6 Ruminal pH

Ruminal pH was measured directly in situ via cannula at 0, 1, 2, 4, 6, and 8 h post-feeding using a calibrated portable pH meter. Mean values were calculated for each animal and period.

### 2.7 Ruminal liquid kinetics

Ruminal liquid passage rate and volume were determined using polyethylene glycol (PEG 6000) as a liquid-phase marker. A dose of 300 g of PEG 6000 (Carbowax 6000; Synth, Brazil), diluted in 500 mL of water, was administered intraruminally through the ruminal cannula immediately after collection of the baseline ruminal fluid sample (0 h), and the ruminal contents were manually mixed to ensure uniform marker distribution. Ruminal fluid samples were subsequently collected at 1, 2, 4, 8, 12, and 24 h after marker administration. Polyethylene glycol concentration was determined according to the method described by Hydén (1956). Liquid passage rate (%/h) was estimated from the slope of the linear regression of the natural logarithm of PEG concentration against sampling time, assuming first-order exponential disappearance of the marker. The quantity of PEG dosed to each animal was divided by the 0 h postdosing intercept of the curve to yield an estimate of fluid volume (Beckett et al., 2021).

### 2.8 Chemical composition of diets

Dry matter, ash, crude protein, starch, neutral detergent fiber (NDF), and acid detergent fiber (ADF) were determined according to AOAC (1990), Hall et al. (2015), Hall et al. (1999) and Van Soest et al. (1991), using standard analytical procedures.

### 2.9 Statistical analysis

Data were analyzed using the MIXED procedure of SAS (SAS Institute Inc., Cary, NC, USA, 2008). The experiment was conducted as a single 4 × 4 Latin square design with a 2 × 2 factorial arrangement of treatments. Each treatment was represented by four observations, one from each animal across the four experimental periods. The statistical model included the fixed effects of dietary NDF level, rumen modulator, and their interaction, while animal and period were included as random effects. For variables measured repeatedly over time (ruminal pH, NH₃-N, SCFA, lactate, and protozoa counts), repeated-measures analysis was performed with sampling time, treatment, and their interactions included in the model. The covariance structure was selected based on the lowest Akaike information criterion. Least squares means were generated and compared using the PDIFF option when significant effects were detected. Model assumptions were evaluated by assessing the normality of residuals. Significance was declared at *P* ≤ 0.05 and tendencies were discussed when 0.05 < *P* ≤ 0.10.

## 3 Results

Dietary NDF concentration significantly affected intake, ruminal function, methane emissions, and feeding behavior. Animals fed 50% NDF diets showed greater dry matter intake (DMI) compared with those fed 12% NDF diets (Table 2). Within the 50% NDF diets, DMI was higher in animals receiving VM compared with those receiving the phytogenic additive, whereas no differences between additives were observed under 12% NDF conditions.

**Table 2:** Effects of a conventional antimicrobial additive strategy (VM; monensin plus virginiamycin) and a plant-derived functional additive (LM+) in diets containing 12 or 50% NDF, methane emissions, feeding behavior, ruminal liquid volume, and ruminal liquid passage rate.

	Treatments							
	50% NDF		12% NDF		*P* value			
	VM	LM+	VM	LM+	SEM	ADD	NDF	ADD x NDF
DMI, kg/d	13.96 ^a^	10.42 ^b^	4.89 ^c^	4.77 ^c^	0.780	0.0247	<.0001	**0.035**
CH_4_, g/d	176.81 ^a^	129.98 ^b^	45.97 ^c^	47.84 ^c^	12.00	0.069	<.0001	**0.049**
CH_4_, g/kg DMI	13.54 ^a^	12.52 ^a^	9.75 ^b^	7.81 ^b^	1.072	0.248	0.0005	0.525
Idling time, min/d	776 ^b^	886 ^b^	1233 ^a^	1226 ^a^	53.56	0.360	<.0001	0.307
Idling time, %	53.66 ^b^	61.66 ^b^	86.00 ^a^	85.00 ^a^	3.790	0.383	<.0001	0.269
Rumination time, min/d	433 ^a^	350 ^a^	76 ^b^	93 ^b^	50.66	0.529	0.0003	0.352
Rumination time, %	30.00 ^a^	24.00 ^a^	5.33 ^b^	6.66 ^b^	3.464	0.549	0.0003	0.341
Eating time, min/d	230 ^a^	203 ^a^	130 ^b^	120 ^b^	25.22	0.488	0.0060	0.749
Eating time, %	16.00 ^a^	14.33 ^a^	9.33 ^b^	8.33 ^b^	1.660	0.4446	0.0051	0.845
Ruminal liquid, L	41.66	42.14	43.80	33.79	4.070	0.287	0.4770	0.245
Liquid passage rate, %/h	4.76^a^	6.09 ^a^	2.98 ^b^	2.64 ^b^	0.930	0.614	0.0270	0.404

Percentages refer to a fixed 24 h observation period. ADD = additive effect; NDF = NDF level effect. SEM = standard error of the mean. Different superscript letters (a–c) within a row indicate significant differences (*P* < 0.05). The 12% and 50% NDF diets represent the forage-free and conventional fiber-based feeding systems, respectively.

Methane emissions (g/day) were higher in animals fed 50% NDF diets compared with 12% NDF diets. A significant diet × additive interaction was observed for methane emissions (g/day). Within the 50% NDF group, VM resulted in greater methane emissions than the phytogenic additive, whereas no differences between additives were observed under the 12% NDF diets. When expressed per unit of DMI, methane emissions remained higher in 50% NDF diets regardless of additive (Table 2).

Animals fed 50% NDF diets spent more time eating and ruminating and less time idle compared with those fed 12% NDF diets, with no effect of additive (Table 2). Ruminal liquid passage rate was greater in animals fed 50% NDF diets, whereas ruminal liquid volume was not affected by either dietary NDF level or additive (Table 2).

No significant three-way interaction between diet, additive, and sampling time was observed for ruminal fermentation variables. Therefore, main effects and two-way interactions are presented.

**Fig. 1:**
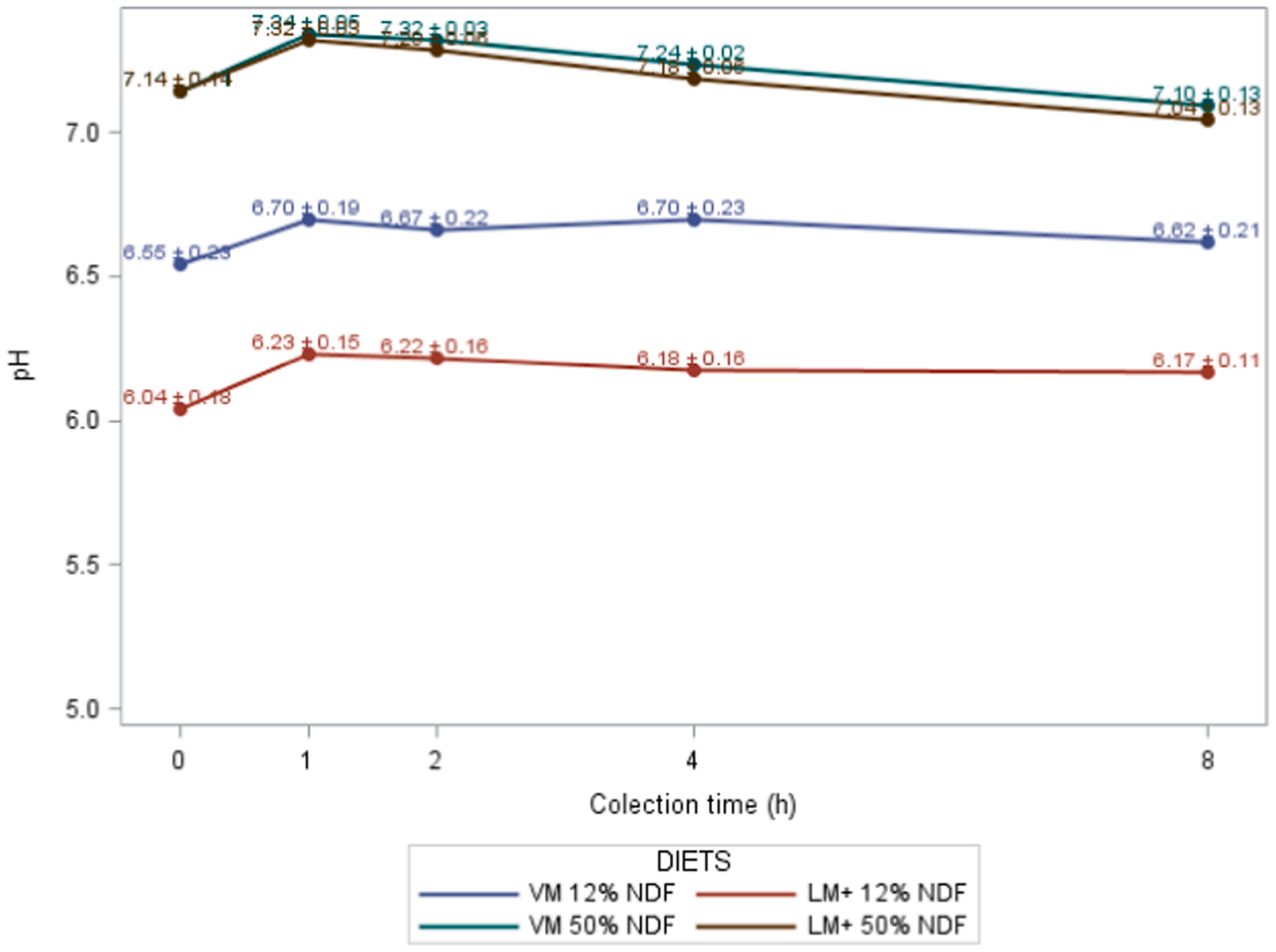
Ruminal pH over time in cows fed diets containing 12 or 50% NDF and supplemented with a conventional antimicrobial additive strategy (VM; monensin plus virginiamycin) or a plant-derived functional additive (LM+). Values are presented as means ± standard error of the mean (SEM).

Ruminal pH was higher in animals fed 50% NDF diets compared with 12% NDF diets. Within 12% NDF diets, VM resulted in higher pH compared with the phytogenic additive, whereas no differences were observed under 50% NDF conditions (Table 3; Fig. 1).

**Table 3:** Effects of a conventional antimicrobial additive strategy (VM; monensin plus virginiamycin) and a plant-derived functional additive (LM+) in diets containing 12 or 50% NDF on ruminal fermentation parameters, pH, ammonia nitrogen, protozoa counts, and volatile fatty acid concentrations.

	Treatments							
	50% NDF		12% NDF		*P* value			
	VM	LM+	VM	LM+	SEM	ADD	NDF	ADD x NDF
pH	7.22 ^a^	7.19 ^a^	6.64 ^b^	6.16 ^c^	0.074	0.0002	<.0001	**0.0012**
NH₃-N, mM	10.00 ^c^	10.43 ^bc^	12.43 ^b^	16.88 ^a^	0.993	0.0021	<.0001	**0.0104**
Protozoa, (× 10⁴ cells/mL)	5.92 ^a^	4.92 ^a^	2.32 ^b^	3.65 ^ab^	0.579	0.7767	**0.0006**	0.0649
Total VFAs, mM	82.41 ^ab^	80.87 ^ab^	70.94 ^b^	94.96 ^a^	6.118	0.0435	0.8109	0.0224
Acetic acid, mM	42.47 ^b^	50.39 ^a^	33.00 ^c^	42.28 ^b^	3.284	0.0021	0.0017	0.8018
Propionic acid, mM	20.04^ab^	16.18 ^b^	20.35^ab^	23.79 ^a^	1.777	0.9121	0.0427	0.0613
Acetate:propionate ratio	2.45 ^b^	3.14 ^a^	1.67 ^c^	2.02 ^b^	0.382	0.0035	<.0001	0.3268
Butyric acid, mM	12.6 ^b^	9.04 ^b^	11.94 ^b^	20.22 ^a^	1.606	0.8690	0.0187	0.0089
Iso-butyric acid, mM	1.23 ^a^	1.20 ^a^	0.92 ^b^	1.20 ^a^	0.060	0.0185	0.0061	0.0045
Valeric acid, mM	2.43 ^b^	0.77 ^c^	1.79 ^bc^	3.90 ^a^	0.466	0.3563	0.4618	0.0030
Iso-valeric acid, mM	3.57 ^a^	3.27 ^a^	2.92 ^b^	3.55 ^a^	0.320	0.1661	0.0743	0.0379
Lactic acid, mM	1.49 ^b^	1.02 ^b^	12.57 ^a^	8.86 ^a^	3.129	0.4198	<.0001	0.3470

VM= monensin plus virginiamycin; LM+= plant-derived functional additive; VFA= volatile fatty acids; NH₃-N= ammonia nitrogen; ADD= additive effect; NDF= NDF level effect; SEM= standard error of the mean. Different superscript letters (a–c) within a row indicate significant differences (*P* < 0.05). The 12% and 50% NDF diets represent the forage-free and conventional fiber-based feeding systems, respectively.

**Fig. 2:**
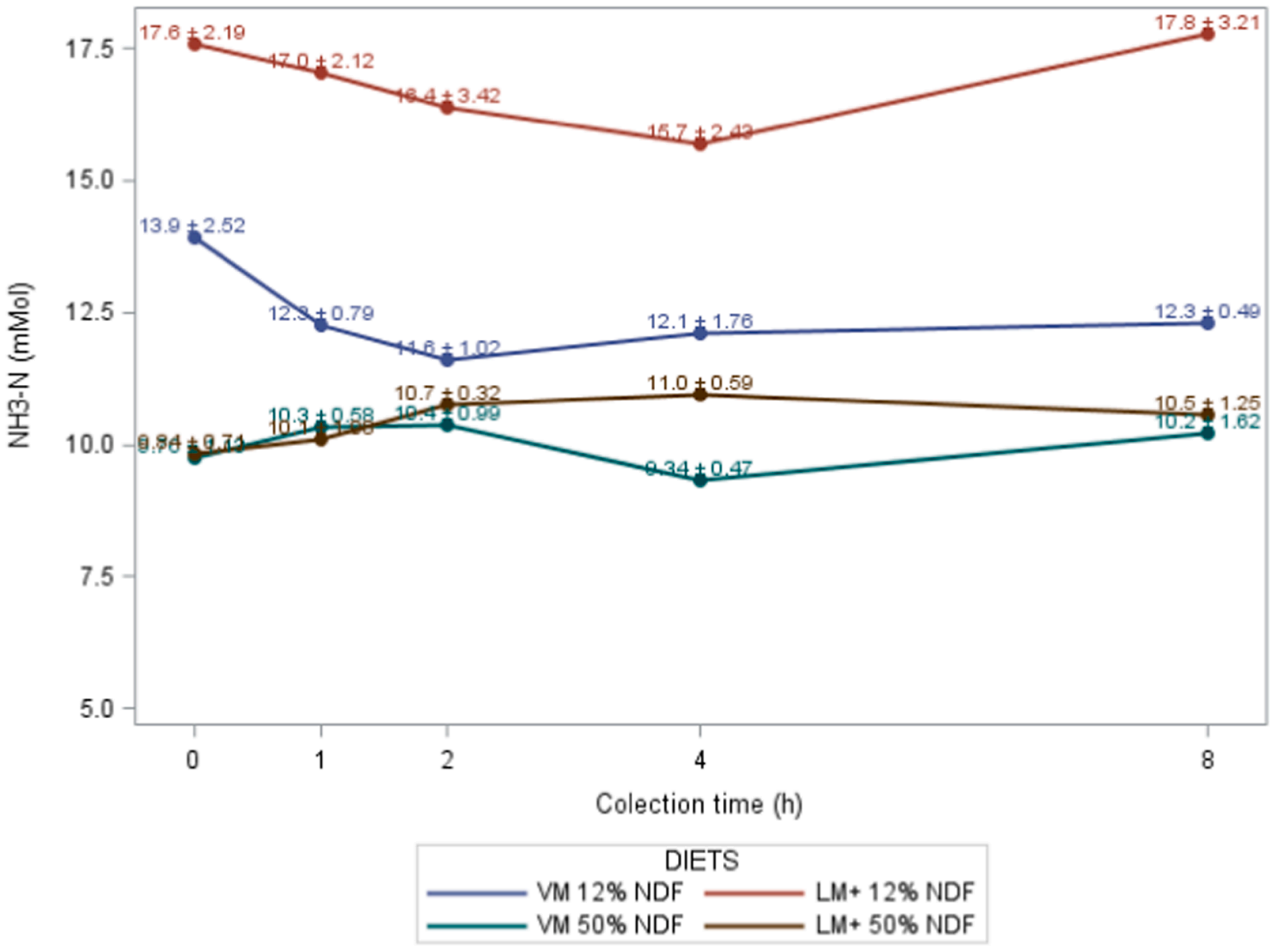
Ammonia nitrogen (NH₃-N) concentration over time in ruminal fluid of cows fed diets containing 12 or 50% NDF and supplemented with a conventional antimicrobial additive strategy (VM; monensin plus virginiamycin) or a plant-derived functional additive (LM+). Values are presented as means ± standard error of the mean (SEM).

Ammonia nitrogen (NH₃-N) concentration was lower in animals fed 50% NDF diets. Within 12% NDF diets, VM reduced NH₃-N concentration compared with the phytogenic additive (Table 3; Fig. 2).

**Fig. 3:**
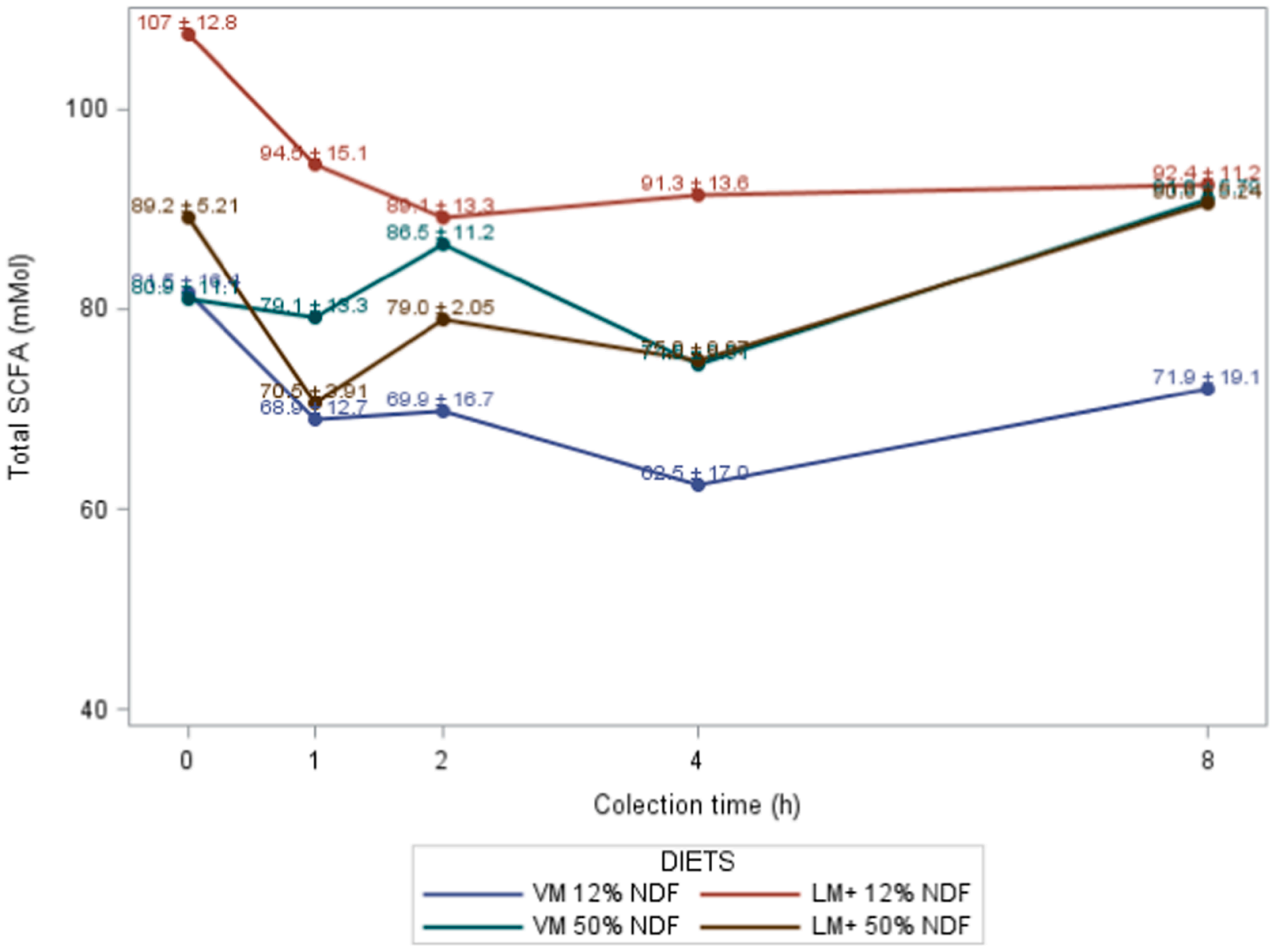
Total short-chain fatty acid (SCFA) concentration over time in ruminal fluid of cows fed diets containing 12 or 50% NDF and supplemented with a conventional antimicrobial additive strategy (VM; monensin plus virginiamycin) or a plant-derived functional additive (LM+). Values are presented as means ± standard error of the mean (SEM).

Total short-chain fatty acid (SCFA) concentration was affected by the interaction between diet and additive. Under 12% NDF conditions, the phytogenic additive increased total SCFA compared with VM, whereas no differences were observed under 50% NDF diets (Table 3; Fig. 3).

Acetate concentration was higher in animals fed 50% NDF diets, and was also greater with the phytogenic additive compared with VM. The acetate-to-propionate ratio followed a similar pattern, being higher in 50% NDF diets and under phytogenic additive supplementation (Table 3).

Propionate concentration showed a diet × additive tendency, with higher values under 12% NDF diets supplemented with the phytogenic additive compared with VM.

**Fig. 4:**
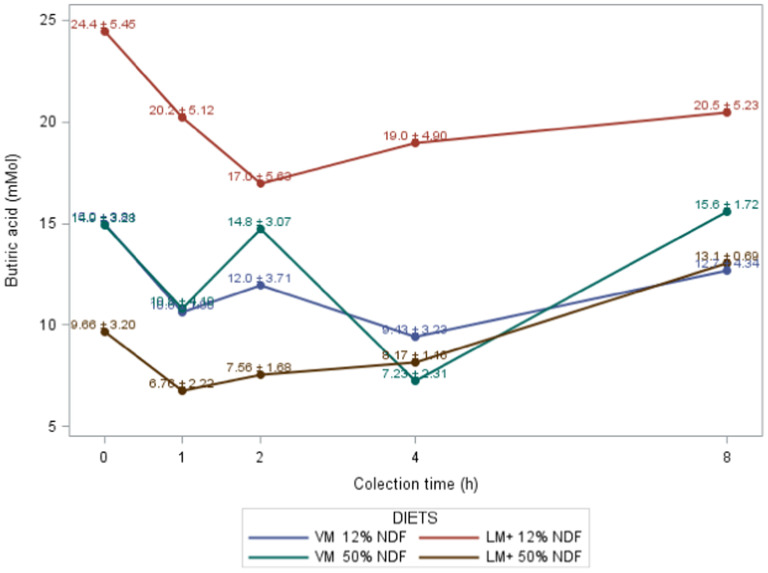
Butyrate concentration over time in ruminal fluid of cows fed diets containing 12 or 50% NDF and supplemented with a conventional antimicrobial additive strategy (VM; monensin plus virginiamycin) or a plant-derived functional additive (LM+). Values are presented as means ± standard error of the mean (SEM).

**Fig. 5:**
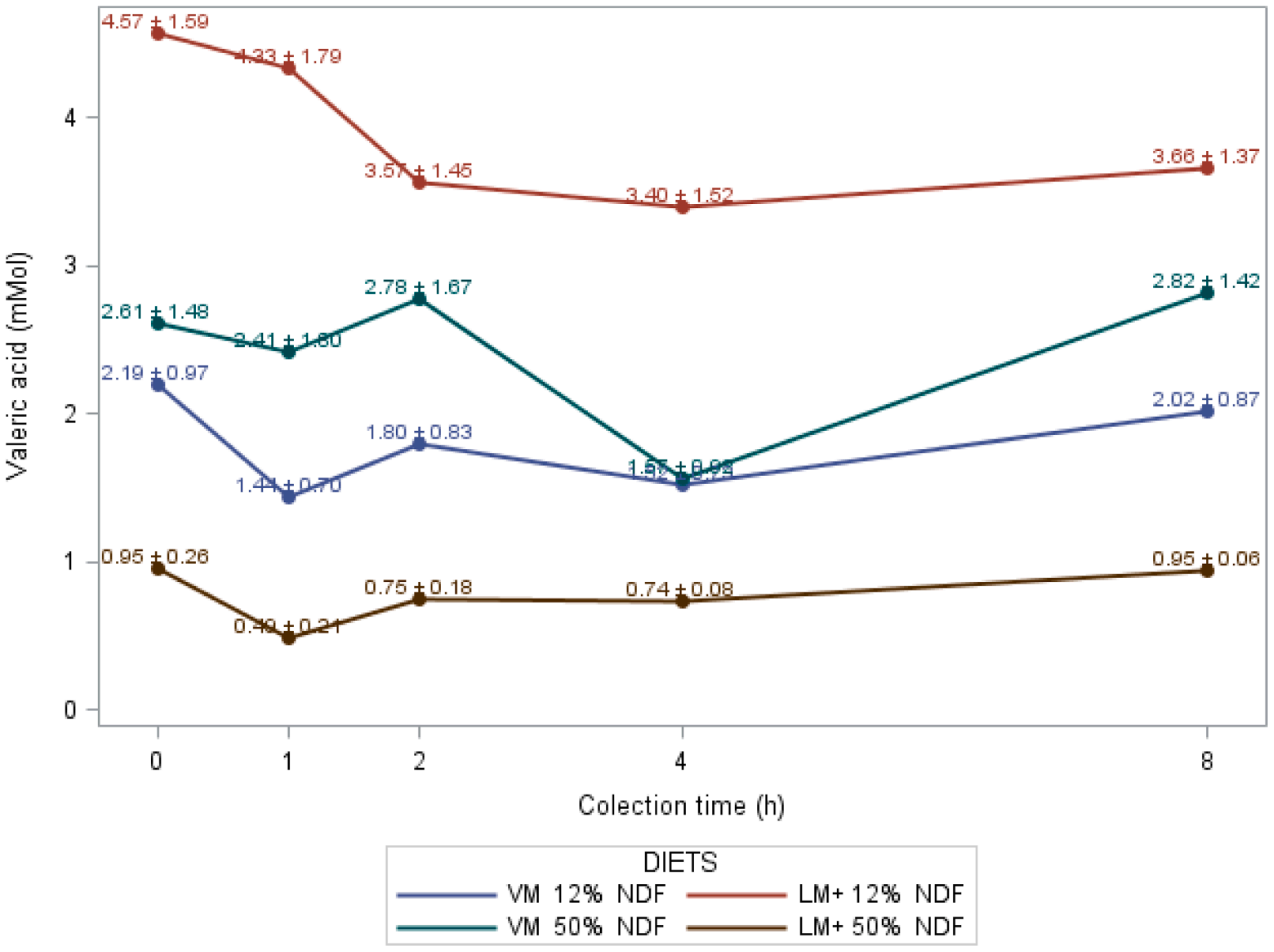
Valerate concentration over time in ruminal fluid of cows fed diets containing 12 or 50% NDF and supplemented with a conventional antimicrobial additive strategy (VM; monensin plus virginiamycin) or a plant-derived functional additive (LM+). Values are presented as means ± standard error of the mean (SEM).

Butyrate concentration was higher in 12% NDF diets supplemented with the phytogenic additive, with no differences observed in 50% NDF diets (Fig. 4). Valerate concentration showed a significant diet × additive interaction, with opposite responses depending on dietary NDF level (Fig. 5).

Iso-butyric and iso-valeric acid concentrations were reduced in 12% NDF diets supplemented with VM compared with other treatments (Table 3).

**Fig. 6:**
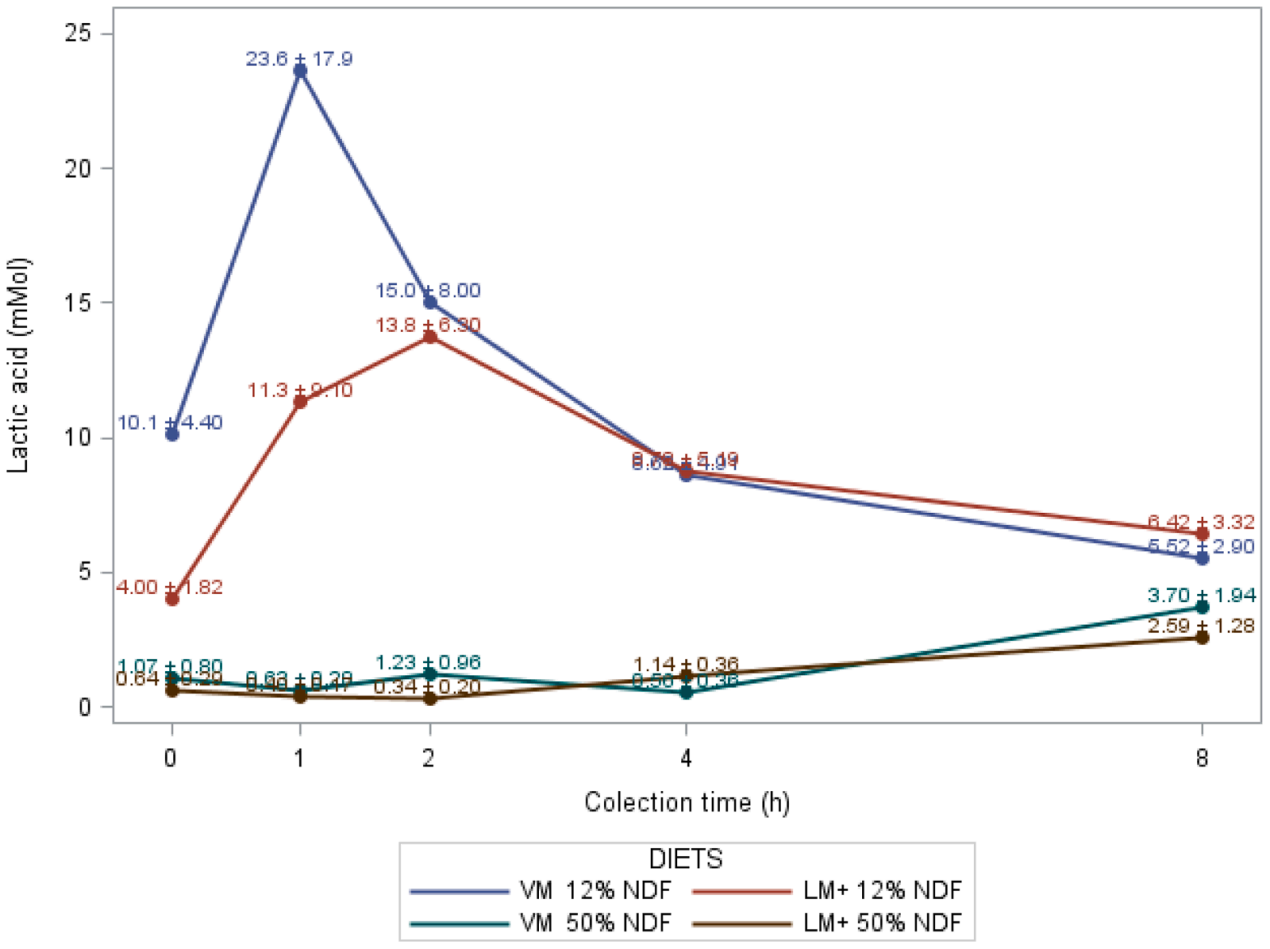
Lactate concentration over time in ruminal fluid of cows fed diets containing 12 or 50% NDF and supplemented with a conventional antimicrobial additive strategy (VM; monensin plus virginiamycin) or a plant-derived functional additive (LM+). Values are presented as means ± standard error of the mean (SEM).

Lactate concentration was higher in 12% NDF diets compared with 50% NDF diets, with no additive effect (Table 3; Fig. 6).

Protozoa counts tended to be lower in 12% NDF diets, with a diet × additive interaction approaching significance (P= 0.06) (Table 3).

Across time-course measurements, ruminal fermentation variables showed expected post-feeding fluctuations; however, no significant diet × additive × time interactions were observed for the variables presented in Fig. 1, Fig. 2, Fig. 3, Fig. 4, Fig. 5, Fig. 6, indicating consistent treatment effects over time.

## 4 Discussion

Dietary fiber concentration was the primary factor influencing intake behavior, ruminal fermentation, and methane emissions. Animals fed 50% NDF diets showed greater dry matter intake, rumination activity, ruminal pH, and liquid passage rate compared with those fed 12% NDF diets. These responses are consistent with the established role of physically effective fiber in stimulating chewing activity, salivary buffering, and ruminal motility, which collectively support ruminal function (Galyean & Defoor, 2003; Nagaraja & Lechtenberg, 2007).

The greater ruminal passage rate observed in high-fiber diets suggests enhanced digesta turnover and ruminal flow dynamics. Similar responses have been reported in cattle receiving forage-based diets, where increased rumination activity is associated with greater fluid dynamics and ruminal stability (Duffield et al., 2008). In contrast, lower intake observed in 12% NDF diets may reflect metabolic feedback mechanisms associated with rapid fermentation of highly fermentable carbohydrates (Allen et al., 2009).

Methane emissions were higher in animals fed 50% NDF diets, consistent with the greater availability of structural carbohydrates favoring acetate-dominated fermentation pathways and hydrogen production. When expressed relative to intake, methane output remained higher in high-fiber diets, indicating that fiber concentration is a key determinant of enteric methane production under the conditions of this study.

The greater methane emissions observed in animals fed 50% NDF diets are consistent with the higher acetate concentrations and acetate-to-propionate ratios detected under these conditions. Previous studies have shown that acetate-dominated fermentation is generally associated with greater hydrogen availability for methanogenesis, whereas propionate formation competes for reducing equivalents, thereby decreasing the availability of substrate for methane formation. Accordingly, the fermentation profile observed under the 50% NDF diets is consistent with metabolic conditions commonly associated with greater enteric methane production. Similar relationships between dietary fiber concentration, acetate-dominated fermentation, and methane production have been consistently reported in ruminants receiving forage-based diets (Hristov et al., 2013; Olijhoek et al., 2021).

Interestingly, the phytogenic additive reduced methane emissions relative to VM under 50% NDF conditions, whereas no additive effect was detected under 12% NDF diets. This response suggests that the efficacy of phytogenic compounds may depend on the prevailing fermentation environment. Essential oils have been proposed to influence methane formation through modifications in ruminal fermentation and microbial activity, although the relative importance of these mechanisms appears to depend on additive composition, dietary characteristics, and ruminal adaptation (Cobellis et al., 2016; Orzuna-Orzuna et al., 2022; Patra & Yu, 2012).

Although lactate concentrations were greater in animals fed 12% NDF diets, ruminal pH remained within physiological ranges throughout the experimental period. This finding suggests that lactate accumulation was limited despite the greater availability of rapidly fermentable carbohydrates, possibly due to an effective balance between lactate production and utilization. This interpretation is consistent with previous observations in adapted cattle receiving highly fermentable diets supplemented with rumen-modulating additives (Ovani et al., 2026).

The absence of additive effects on lactate concentration further suggests that dietary fiber concentration exerted a stronger influence on lactate dynamics than the rumen modulators evaluated in the present study. These results reinforce the importance of dietary adaptation and ruminal resilience in maintaining fermentation stability under forage-free feeding conditions.

Protozoal counts tended to be lower in animals fed 12% NDF diets, although the interaction between dietary fiber concentration and additive approached, but did not reach, statistical significance. This tendency is consistent with previous observations indicating that high-concentrate diets may reduce ruminal protozoal populations through decreases in ruminal pH and changes in substrate availability.

Because ruminal protozoa contribute to hydrogen transfer within the ruminal ecosystem and are closely associated with methanogenic archaea, the lower protozoal counts observed under low-fiber conditions may be consistent with one of the mechanisms previously proposed to explain reduced methane emissions (Newbold et al., 2015). However, given the limited magnitude of the response and the absence of direct measurements of methanogenic populations, this potential mechanism remains speculative and should be interpreted with caution.

Dietary fiber level also modulated the response to rumen modulators. The phytogenic additive and VM showed distinct effects depending on dietary context, supporting the hypothesis of diet-dependent efficacy of rumen modulators. Under 12% NDF conditions, VM reduced ruminal ammonia nitrogen concentrations, consistent with its known inhibitory effects on deamination and proteolytic microbial populations (Russell & Strobel, 1989). In contrast, the phytogenic additive altered fermentation end-products without consistently reducing ammonia nitrogen, suggesting a different mode of action on ruminal microbial activity.

Total SCFA concentration was increased by the phytogenic additive under 12% NDF conditions, suggesting altered fermentation patterns under high-concentrate diets (Fig. 3). Similar variability in responses to essential oils and phytogenic compounds has been widely reported, with effects strongly dependent on diet composition, dose, and ruminal environment (Benchaar et al., 2008; Cobellis et al., 2016; Hristov et al., 2013; Olijhoek et al., 2021). Such inconsistencies highlight the complexity of predicting microbial responses to plant-derived additives in vivo.

Changes in individual volatile fatty acid profiles further support the diet-dependent modulation of ruminal fermentation. Increased butyrate and valerate concentrations under the 12% NDF diets supplemented with the phytogenic additive are consistent with shifts in fermentation pathways previously reported for phytogenic compounds under high-concentrate feeding conditions (Patra and Yu, 2012). However, the magnitude and direction of these responses varied across dietary treatments, reinforcing the importance of dietary context in determining the fermentation response to rumen modulators.

Differences between studies in the literature are partially explained by methodological variability, particularly between in vitro and in vivo systems. In vitro systems often exaggerate microbial responses and do not capture host-mediated factors such as feed intake regulation, ruminal turnover, and physiological adaptation, which are critical determinants of fermentation outcomes in vivo (Cobellis et al., 2016; Patra & Yu, 2012).

Overall, the present results indicate that phytogenic additives may act as effective rumen modulators under specific dietary conditions, particularly in high-concentrate feeding systems; however, their effects are inconsistent and strongly dependent on dietary fiber level and ruminal environment. VM, in contrast, showed more consistent effects on ruminal nitrogen metabolism, particularly under low-fiber conditions.

These findings reinforce the concept that dietary composition is a primary driver of ruminal fermentation dynamics and determines the magnitude and direction of responses to rumen modulators in cattle systems.

## 5 Conclusions

Dietary NDF concentration was the major source of variation, but several fermentation responses were modulated by significant diet × additive interactions. Animals fed 50% NDF diets exhibited greater dry matter intake, rumination activity, ruminal pH, and methane emissions, whereas 12% NDF diets promoted lower intake and altered ruminal fermentation patterns, including increased short-chain fatty acid production and reduced methane emissions per unit of intake. Responses to rumen modulators were dependent on dietary fiber level. Under forage-free conditions, the conventional antimicrobial strategy (monensin plus virginiamycin) reduced ruminal ammonia nitrogen concentrations, suggesting improved ruminal nitrogen utilization under this dietary context. In contrast, the phytogenic additive modulated ruminal fermentation end-products and increased total short-chain fatty acid production under high-concentrate conditions, while showing variable effects under different dietary contexts. These results demonstrate that the effects of rumen modulators are strongly dependent on dietary composition, particularly neutral detergent fiber concentration, highlighting the importance of considering diet–additive interactions when evaluating strategies to modulate ruminal fermentation and methane emissions in cattle.

## CRediT authorship contribution statement

**Geraldo Balieiro Neto:** Writing – review & editing, Writing – original draft, Validation, Supervision, Project administration, Methodology, Investigation, Formal analysis, Data curation, Conceptualization. **Alexandre Berndt:** Supervision, Methodology, Investigation, Formal analysis, Data curation, Conceptualization. **Cristina Maria Pacheco Barbosa:** Writing – original draft, Supervision, Methodology, Investigation, Formal analysis, Data curation, Conceptualization. **Carlos Eduardo Kolb Maynardes Araujo de Campos Jordao:** Writing – original draft, Supervision, Methodology, Investigation, Formal analysis, Data curation, Conceptualization. **Gabriela Aferri:** Writing – original draft, Visualization, Project administration, Methodology, Investigation, Formal analysis, Data curation, Conceptualization. **Leandro Samia Lopes:** Writing – review & editing, Writing – original draft, Supervision, Resources, Methodology, Investigation, Funding acquisition, Formal analysis, Data curation, Conceptualization.

## Declaration of competing interest

The authors declare that they have no known competing financial interests or personal relationships that could have appeared to influence the work reported in this paper.
